# Emotion recognition based on group phase locking value using convolutional neural network

**DOI:** 10.1038/s41598-023-30458-6

**Published:** 2023-03-07

**Authors:** Gaochao Cui, Xueyuan Li, Hideaki Touyama

**Affiliations:** Graduate School of Engineering, Toyama Prefectural University, Imizu, 9390398 Japan

**Keywords:** Engineering, Attention, Emotion

## Abstract

Electroencephalography (EEG)-based emotion recognition is an important technology for human–computer interactions. In the field of neuromarketing, emotion recognition based on group EEG can be used to analyze the emotional states of multiple users. Previous emotion recognition experiments have been based on individual EEGs; therefore, it is difficult to use them for estimating the emotional states of multiple users. The purpose of this study is to find a data processing method that can improve the efficiency of emotion recognition. In this study, the DEAP dataset was used, which comprises EEG signals of 32 participants that were recorded as they watched 40 videos with different emotional themes. This study compared emotion recognition accuracy based on individual and group EEGs using the proposed convolutional neural network model. Based on this study, we can see that the differences of phase locking value (PLV) exist in different EEG frequency bands when subjects are in different emotional states. The results showed that an emotion recognition accuracy of up to 85% can be obtained for group EEG data by using the proposed model. It means that using group EEG data can effectively improve the efficiency of emotion recognition. Moreover, the significant emotion recognition accuracy for multiple users achieved in this study can contribute to research on handling group human emotional states.

## Introduction

**Figure 1:**
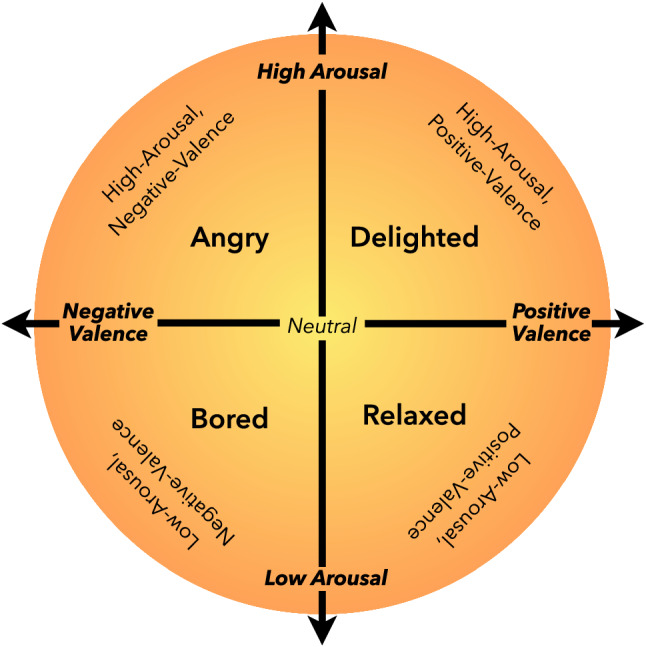
Arousal and valence emotion model.

Machine-learning-based emotion recognition technology plays a crucial role in advanced human–computer interaction^1^. Figure 1 shows the valence-arousal model commonly used in emotion recognition research. This technology allows machines to perceive human emotional changes and adjust the human–computer interaction system accordingly to improve its work efficiency. For example, knowing users’ emotions when they are watching videos can enable recommendation systems to better understand their preferences. The recommendation system can adjust the type of recommended videos shown based on the emotional feedback of a user^2,3^.

In previous research, both non-physiological and physiological signals-based emotion recognition methods have been commonly used. In non-physiological signals-based emotion recognition, emotions have often been identified using voice and facial. In speech-based emotion recognition, Markov models and artificial neural networks have been used to analyze emotions^4,5^. Their results showed that utterances and window size selection affect the performance of an emotion recognition system^6^. In facial expression-based emotion recognition, features are extracted from static, dynamic, or geometric facial characteristics and used as inputs for the model^7–9^.

In physiological signals-based emotion recognition, vital signs such as heart rate^10^, respiration^11^, electroencephalogram (EEG)^12^ are recorded to identify emotions. EEG has significant advantages such as ease of use, cost effectiveness, mobility, and fewer physical constraints, and it can also record the underlying human brain activity. Therefore, EEG has strong reliability for emotion recognition^13^. In contrast, facial expressions and speech can be easily affected by human subjectivity and manipulation. Moreover, owing to its high temporal resolution, EEG can provide unique information regarding neuronal oscillations and cross-frequency coupling, that is, interactions among different frequency bands.

In research^14^, by calculating the differential entropy (DE) features extracted from Beta and Gamma bands of EEG to recognize the different emotions and the accuracies were over 70%. In research^15,16^, event-related potentials (ERPs) were verified to correlate with valence and arousal because ERPs reflected the underlying cognitive process, including emotion. Hence, characteristics of ERP components, e.g., P100, N100, N200, P200, P300, were also taken into studies for emotion recognition and the accuracies were over 79%. Brain network indexes also be used for emotion recognition. The estimation and construction of brain networks based on EEG channels are achieved by calculating correlation or coherence , including Pearson’s correlation coefficient (PCC), mutual information^17^, etc. Based on the specific criteria to calculate or evaluate the relationship between EEG features and the target emotions to find important features for emotion recognition. The important features can be reserved for further model design,and others will be abandoned^18^ such as the Chi-squared test-based feature extract method. These EEG features are also used for emotion recognition in deep learning network. In the research^19^, PCC featured images were used in a deep convolutional neural network with SVM and ensemble learning for emotion recognition. The maximum recognition accuracy were 78.22% on valence and 74.92% on arousal.

In EEG-based emotion recognition research, through auditory and visual stimuli containing different emotions, the relevant time-domain or frequency-domain feature components are extracted from the EEG of an individual to identify emotions.The emotions can be evoked by making them watch a certain video or listen to a certain piece of music^14,20^. And based on participants’ dependent or independent emotional states that caused neural activities in critical brain areas and frequency bands, six different features were extracted to input stable EEG patterns for emotion recognition. For the DEAP dataset, Zheng’s research^21^ achieved an accuracy of 69.67% for emotion recognition on four valance/arousal states. In Heraz’s research^22^, three features were extracted based on the DEAP dataset for EEG analysis, and the trained emotion recognition model achieved a 73.5% accuracy for valance-arousal state classification. In Albraikan’s research^23^, two neural network classifiers were trained to perform a two-class emotion classification on the DEAP dataset, and they achieved an accuracy of 71%. In Lin’s research^24^, the results showed that combining classifiers, such as a support vector machine and hidden Markov model, based on time and discrete wavelet domain features can improve emotion recognition accuracy.

Although these methods obtained good experimental results, they have a common limitation: the univariate EEG features extracted using these methods lack spatial correlation between electrodes. The phase locking value (PLV) method is commonly used for calculating the correlation between electrodes. The PLV is a statistic that can be used to investigate EEG data for task-induced changes in the long-range synchronization of neural activity. The PLV method can separate the phase component from the amplitude component in EEG recordings, and consequently is a suitable choice for exploring synchronous neuronal activities independently of amplitude changes. This characteristic spatial and temporal shifts in synchronization have strongly related to emotion activity. Applying these spatiotemporal characteristics to the deep learning neural network can make the neural network more quickly and easily capture the differences between different emotions. By using PLV for EEG analysis, more characteristic information can be preserved between the electrodes and emotion recognition can be guaranteed. An innovation of this paper is the use of the PLV calculated based on group EEG to identify emotions. In this paper, a PLV-based emotion recognition CNN network model to classify high/low arousal and high/low valence emotions in the DEAP dataset is proposed. Furthermore, the performances of the model for recognizing emotions from group and individual data based on the PLV adjacency matrix were compared.

## Results

### PLV analysis

**Figure 2:**
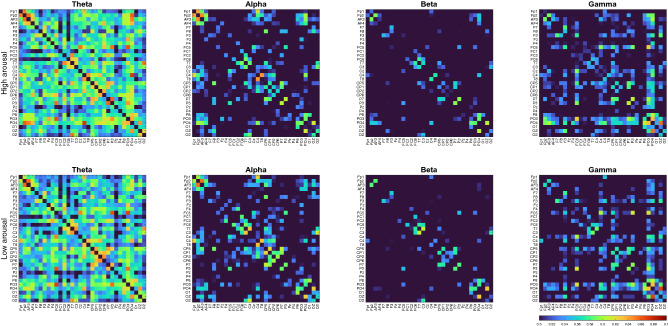
Average PLV value of arousal emotion.

**Figure 3:**
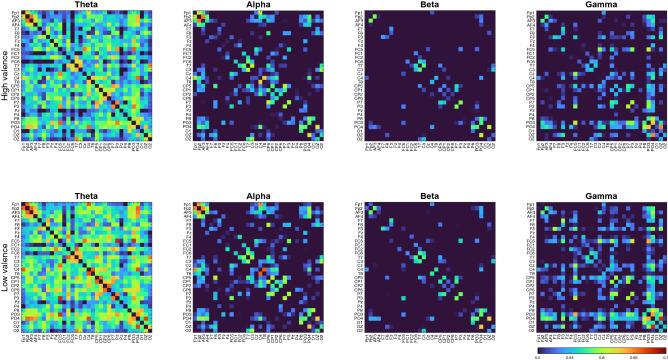
Average PLV value of valence emotion.

Figures 2 and 3 show the average PLV adjacency matrices calculated for arousal and valence emotions, respectively. In Fig. 2, the first and second rows show the average PLV adjacency matrices of high and low arousal, respectively, for different EEG frequency bands.

At the same scale, the differences between individual bands are significant. As shown in Fig. 2, the PLV of the theta band is much higher than those of other bands, whereas the beta band has the lowest PLV. This indicates that the theta frequency bands had higher synchrony when participants were watching the videos. The same results can also be observed in the valence analysis, as shown in Fig. 3.

In the high-arousal state results shown in Fig. 2, each frequency band has a higher PLV than the low-arousal state. However, in the high-valence state results, the PLV is lower than that in the low-valence state in all frequency bands (Fig. 3).

### CNN prediction based on individual and group PLV

**Figure 4:**
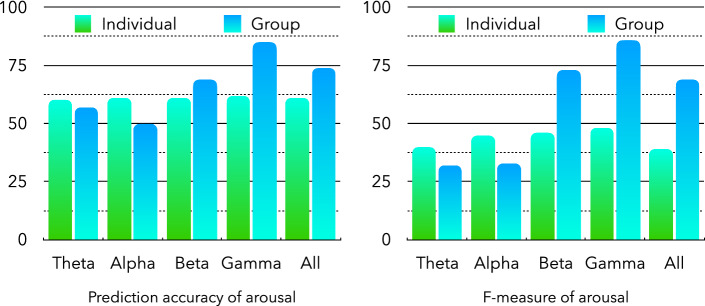
Accuracy and F-measure of arousal emotion prediction.

**Figure 5:**
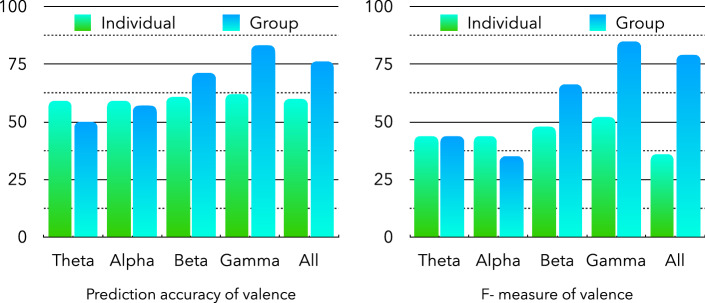
Accuracy and F-measure of valence emotion prediction.

Figures 4 and 5 show the emotion prediction accuracies of the proposed CNN network for individual and group PLV matrices based on different frequency bands. For the prediction of arousal state based on the group PLV matrix (Fig. 4), the gamma band shows the best prediction accuracy of 83%, whereas the alpha and theta bands show lower prediction accuracies of approximately 50–57%. However, based on the individual PLV matrices, all frequency bands show similar prediction accuracies of approximately 60%. Furthermore, in the theta and alpha bands, the individual PLV-based prediction accuracy is higher than that of the group PLV-based prediction. However, in the beta and gamma bands, the group PLV-based prediction accuracy is better than that of the individual PLV-based prediction. The F-measures (Fig. 4) also show the same results.

The valence emotion predictions (Fig. 5) based on individual and group PLV also show similar results. For the group PLV-based valence predictions, the gamma band shows the best prediction accuracy of 85%, whereas the theta band shows the lowest accuracy of 50%. For the individual PLV-based valence predictions, the accuracies of all the frequency bands range from 59 to 62%.

**Figure 6:**
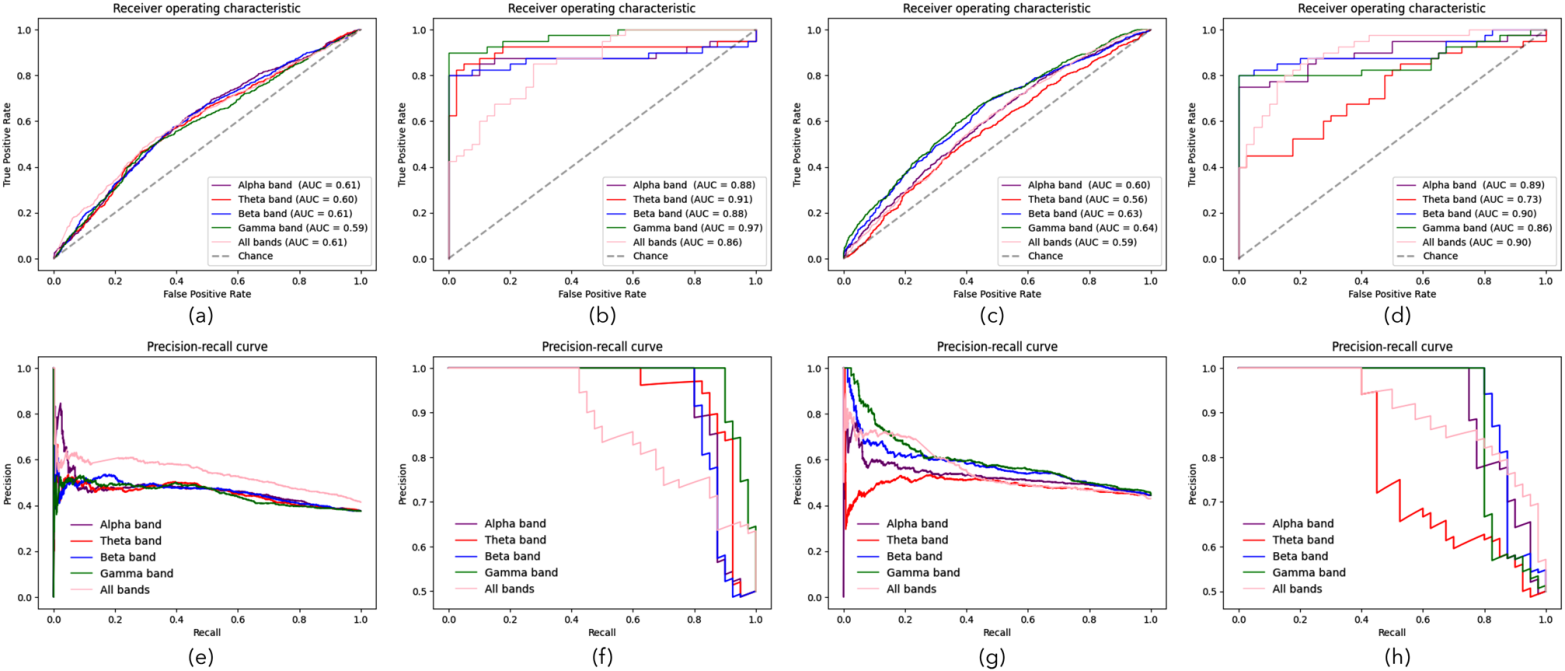
ROC and PR curve of arousal/valence emotion prediction based on individual and group PLV: (**a**),(**e**) arousal emotion prediction based on individual PLVs, (**b**),(**f**) arousal emotion prediction based on group PLVs, (**c**),(**g**) valence emotion prediction based on individual PLVs, (**d**),(**h**) valence emotion prediction based on groups PLVs.

Table 1 is the results of precision and recall rates of arousal/valence emotion prediction based on individual and group PLV. It also shows that the group PLV-based arousal and valence emotion prediction have higher precision and recall rates than individual PLV-based prediction. Receiver operating characteristic (ROC) curve and precision-recall (PR) curve are also calculated for shown the prediction model performance which were shown in Fig. 6. In Fig. 6(a, e) and Fig. 6(b, f) are the ROC and PR curve of arousal emotion prediction based on individual and group PLVs. Fig. 6(e, g) and Fig. 6(d, h) are the ROC and PR curve of of valence emotion prediction based on individual and group PLVs. In the ROC and PR curve, different color represent different frequency bands. The result shows that the proposed prediction model based on CNN have similar performance on the each frequency bands of individual PLV. On the beta and gamma frequency bands of group PLV, there are higher precision and recall rates than other frequency bands. The group PLV-based arousal/valence emotion prediction results also have higher area under curve (AUC)values on each frequency bands than individual PLV-based analysis results.

**Table 1:** Precision and recall rates of valence/arousal prediction based on individual and group PLV.

Condition	Theta	Alpha	Beta	Gamma	All
Individual PLV-based valence precision	0.51	0.53	0.56	0.55	0.54
Individual PLV-based valence recall	0.55	0.47	0.44	0.49	0.41
Group PLV-based valence precision	0.61	0.90	0.81	0.93	0.84
Group PLV-based valence recall	0.65	0.67	0.95	0.91	0.95
Individual PLV-based arousal precision	0.52	0.52	0.54	0.54	0.54
Individual PLV-based arousal recall	0.43	0.43	0.43	0.45	0.51
Group PLV-based arousal precision	0.51	0.47	0.70	0.80	0.75
Group PLV-based arousal recall	0.65	0.85	0.90	0.90	0.90

## Discussion

To analyze the difference in brain activity of participants in high and low arousal/valence emotion, in this paper, EEG-based PLV were analyzed, and a CNN-based deep learning network was proposed to recognized emotions using individual and group PLV.

In the PLV results, a difference between high and low arousals was observed in each frequency band, and some specific areas showed significant differences. For example, in the results of arousal emotion, when participants were in a high-arousal emotional state, the synchrony between PO3, PO4, P8, and Fp2, AF3, AF4 was significantly higher than that in the low-arousal state.

Moreover, the synchrony of some regions showed an opposite trend. For example, when participants were in a high-arousal emotional state, the synchrony between C3 and T7 was lower than that in the low-arousal emotional state. This indicates that when participants are in different emotional states, various brain regions respond differently, and these differences can be used as features for emotion classification. In the results of valence emotion, when participants were in the low-valence emotional state, the synchrony of each channel in the brain was higher than that in the high-valence emotional state. For example, the synchrony between PO3, PO4, P8 and Fp2, AF3, AF4 was significantly higher in the low-valence emotional state.

In the recognition experiments of arousal and valence emotional states based on the proposed CNN network, the prediction accuracies of different frequency bands were mainly compared between individual and group data-based PLV. The recognition results of the arousal emotional state based on individual data showed that all frequency bands obtained almost the same accuracy. However, the results of arousal emotional state based on group data showed that the accuracy rates of gamma frequency bands were significantly higher than those of other frequency bands, and the recognition accuracy rates of high-frequency bands were higher than those of low-frequency bands. The results of precision and recall rates in Table 1 also verified this conclusion. The group PLV-based precision and recall rates in Beta and Gamma frequency bands are higher than other frequency bands based on individual PLV.

Similar results were reported in a recognition experiment of valence emotion states. The noise in the high-frequency bands of the EEG data were lower than those in the low-frequency bands, and the individual differences were also reduced based on the average calculation of the group PLV. Therefore, the recognition accuracy rates were significantly improved in the high-frequency bands of the EEG. The ROC and PR curve results also report that proposed model have better performance in the high-frequency bands.

Based on the experiment results, we can know that when the participants are in a high arousal/valence emotion state, the synchronicity between forehead and back occiput of the brain show relatively strong activity, and at the same time, the synchronicity of middle areas decreases with the increase of excitement.

In this study, we constructed a deep learning model to recognize the emotions of the participants using PLV matrix which was calculated based on the EEG signal. This paper studies a generalization problem on emotion recognition based on the DEAP dataset with a small amount of data. In future research, we will increase the amount of dataset and improve the deep learning network to further improve the accuracy of classification and the number of emotion types identified, such as high valence—high arousal, high valence—low arousal, low valence—high arousal, low valence—low arousal 4-class recognition. Based on the increase in the amount of data and the improvement of the deep learning network, this emotion recognition model could be generalized to cross-user or and user-independent models.

## Conclusions

In this study, we analyzed the DEAP dataset containing high valence, low valence, high arousal and low arousal based on the PLV method. The analysis results show that there are significant differences between high valence and low valence in the beta frequency band and gamma frequency band. And the same difference also appears in the PLV matrix of high arousal and low arousal.

Furthermore, a CNN deep learning network based on PLV matrix for emotion recognition is also constructed. Using this CNN network, two dimensions of high valence/low valence and high arousal/low arousal are respectively analyzed for emotion recognition based on the individual PLV matrix data set and the group PLV matrix data set. The analysis results show that the emotion recognition accuracy based on group data is higher than individual data. This means that the group PLV data contains more effective features that can be used to predict the state of the group, and it also improves the possibility of group sentiment prediction being applied in the neural marketing and other fields.

## Methods

### EEG recording of DEAP

DEAP dataset is a large collection of physiological signals recorded with the aim of developing emotion recognition systems^25^. This dataset includes 32 participants’ (16 male and 16 female) physiological signals such as EEG. The average age of participants is 26.9 years old with age ranging between 19 and 37. Before the experiment began, all the participants signed a consent prior to experiments and filled out the questionnaires. And all the participants were asked to understand a set of instructions for recording the self-assessment reports.

**Figure 7:**
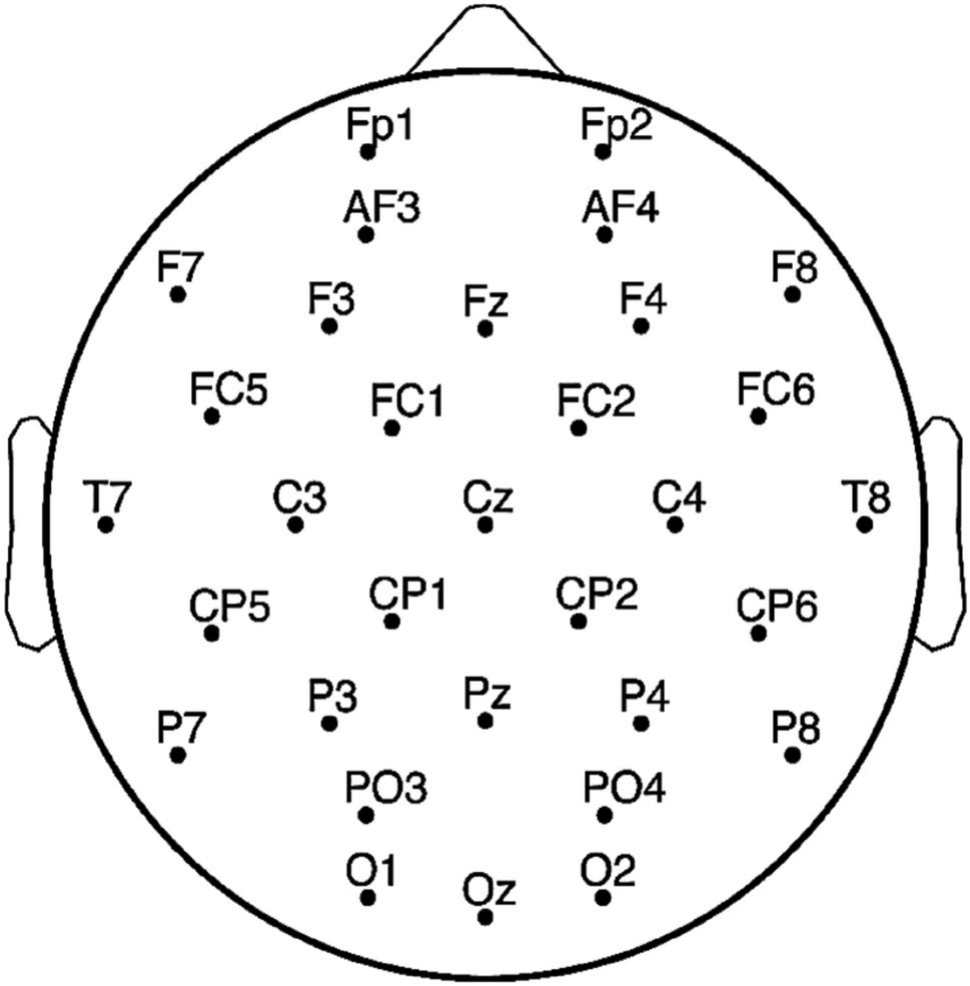
EEG channels location.

The participants were asked to watch 40 videos and rate the 40 videos on valence, arousal, dominance, liking and familiarity from 1 to 9 after watching each video. In the DEAP dataset, EEG was recording from 3 s before video started until the video over, 63 s totally. The EEG electrodes were setting 32 channels based on 10–20 system of electrode placement (Fig. 7) and the sampling frequency is 128 Hz in the DEAP which was downscaling from 512 Hz in original dataset. Therefore, the EEG dataset of 32 $\times$ 40 $\times$ 32 $\times$ 8064 (participants $\times$ videos $\times$ channels $\times$ EEG sampling points) was recorded in DEAP. The DEAP datasets analyzed during the current study are available in https://www.eecs.qmul.ac.uk/mmv/datasets/deap/readme.html.

#### EEG data preprocessing and calculation of PLV adjacency matrix

Because the first three seconds of the 63 s in EEG recording were the time to prepare to watch the video, so it will be removed, and 60 s EEG data were used for data analysis in our experiments. The 60 s of EEG data will be divided into 20 segments in every three seconds. Based on 32 participants and 40 videos, the EEG data size will be 32 $\times$ 40 $\times$ 32 $\times$ 20 $\times$ 384 (participants $\times$ videos $\times$ channels $\times$ segments $\times$ EEG sampling points) after preprocessing. Moreover, according to the rating of the video, videos with a rating higher than 4.5 will be marked with High Arousal/Valence, and the others will be marked with Low Arousal/Valence as labels.

**Figure 8:**
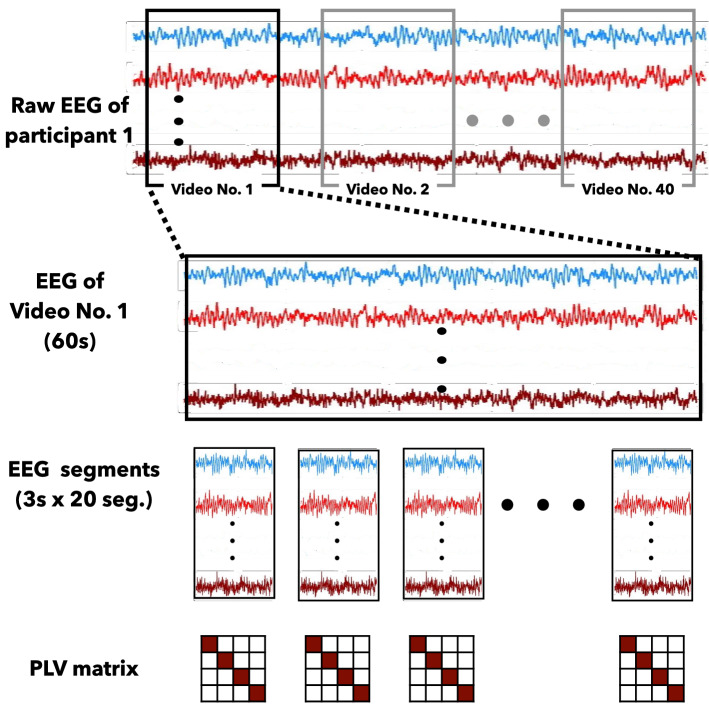
The example of EEG processing.

To prepare the training data and testing data for deep learning, the PLV matrixes of theta rhythm (4–8 Hz), alpha rhythm (8–13 Hz), beta rhythm (13–30 Hz) and gamma rhythm (30–45 Hz) will be calculated for each EEG data segment. Using the PLV analysis method, 32 $\times$ 40 $\times$ 20 (participants $\times$ videos $\times$ EEG segments) of PLV matrixes in each EEG rhythm will be obtained based on the preprocessing EEG dataset. Eventually, 25600 PLV matrixes will be calculated for model training and testing analysis in the individual EEG data-based emotion recognition. In the group EEG data-based emotion recognition analysis, the PLV adjacency matrices of 32 participants in the same video will be averaged into one matrix to represent the entire group’s (32 participants) feelings about this video and 800 PLV matrixes will be calculated as the input data for model training and testing. Figure 8 shown the EEG preprocessing and PLV analysis structure.

#### Phase locking value method

The PLV is an analysis method used for calculating interactions between signals. It provides the information regarding phase coupling between two EEG signals. The amplitude $A(t)$ and the instantaneous phase $\phi (t)$ of a signal $s(t)$ can be estimated using the Hilbert transform $H \{ \cdot \}$as follows:

1 $$\begin{aligned} z(t)=s(t)+i\cdot H\{s(t)\}=A(t)\cdot e^{i\cdot \phi (t)} \end{aligned}$$

The analytic signal $z(t)$ can be considered as an embedding of the one dimensional time-series in the 2D complex plane.

2 $$\begin{aligned} \phi (t)=arctan\left( \frac{Im\{ z(t)\} }{Re\{ z(t)\}} \right) =arctan\left( \frac{H \{ s(t)\} }{ s(t)} \right) , \phi \in [-\pi , \pi ] \end{aligned}$$

The phase synchronization is defined as the locking of phases of two oscillators:

3 $$\begin{aligned} \phi _{12} (t)=\phi _{1} (t)-\phi _{2} (t) \end{aligned}$$

where $\phi _1 (t)$ and $\phi _2 (t)$ denote the phases of the oscillators, and $\phi _{12} (t)$ is defined as their relative phase.

The PLV is defined as:

4 $$\begin{aligned} PLV=\left( \left[ \frac{1}{N} \sum ^{N-1}_{j=0} sin(\phi _{12}(j\Delta t)) \right] ^2+\left[ \frac{1}{N} \sum ^{N-1}_{j=0} cos(\phi _{12}(j\Delta t)) \right] ^2\right) ^{\frac{1}{2}} \end{aligned}$$

where $i$ is the imaginary unit; $N$ is the total number of samples; $\phi _{12}$ denotes the relative phase of two signals, and $\Delta t$ denotes the time between the successive samples $j$ from 1 to N-1.

The PLV is bound from 0-1, where 0 indicates unsynchronized phases and 1 indicates a constant phase difference, i.e., perfect synchronization of signals. A decrease in the PLV between two signals indicates a loss of synchronization between them.

#### Proposed deep learning network based on CNN

In recent years, a surge of deep learning-related methods such as cascade of CNN has been used for EEG signal analysis because the deep learning architectures could capture both spatial and temporal information based on the EEG signal which recorded from bio-potentials over time.

**Figure 9:**
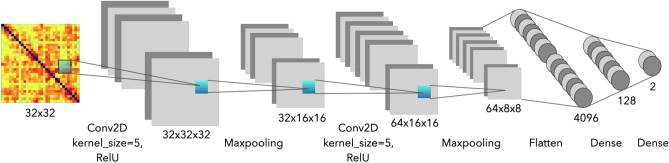
The structure of proposed network model.

The structure of proposed CNN-based network model is shown in Fig. 9. In this research, PLV-based emotion recognition using CNN was performed by fed the adjacency matrices from pre-processing step to first convolution layer for extracting features. In the first convolution layer, filters size was 32 and the kernel size was 5. The input data size was 32*32*1, same with PLV matrix. The parameter of padding was setting as same. The output adjacency matrices were passed to Batch Normalization layer. By using batch normalization to improve the gradient convergence speed, speed up the training speed of the model, reduce the difference between features, and alleviate the problem of gradient disappearance. The output adjacency matrices were passed to max pooling layer to find the underlying features of PLV matrices. After Maxpooling layer, the data will passes through the Dropout layer, and repead the deep learning process from convolution layer to Dropout layer to improve the prediction accuracy of the neural network. In the second convolution layer, filters size was 64 and the kernel size was 5. The parameter of padding was setting as same. The output data will be converted into one-dimensional data by the Flatten layer. After passing through the activation function of the Dense layer, the softmax function of the Dense layer will make classification. Here, the activation function in Dense layer is ReLU and the hyperparameters of the model were set to dropout rate 0.4, batch size 32, and learning rate 0.0001. After multiple convolution layer, Batch Normalization layer and Maxpooling layer process, the depth of the network can be increased, and useful features and local information can be better captured from the PLV adjacency matrix. Moreover, it could also further improve network performance and reduce over-fitting.

## Data Availability

DEAP datasets are publicly available dataset for emotion research. Everyone could download this datasets from https://www.eecs.qmul.ac.uk/mmv/datasets/deap/readme.html.
